# Administration of HES in elderly patients undergoing hip arthroplasty under spinal anesthesia is not associated with an increase in renal injury

**DOI:** 10.1186/s12871-017-0320-8

**Published:** 2017-02-21

**Authors:** Yuanyuan Zhang, Yonghao Yu, Junya Jia, Wenli Yu, Rubin Xu, Licheng Geng, Ying Wei

**Affiliations:** 1 Department of Anaesthesiology, Tianjin Medical University General Hospital, Anshan Road 154#, Heping District, Tianjin, CN 300052 People’s Republic of China; 2 Tianjin Research Institute of Anaesthesiology, Anshan Road 154#, Heping District, Tianjin, CN 300052 People’s Republic of China; 3 Department of Nephron, Tianjin Medical University General Hospital, Anshan Road 154#, Heping District, Tianjin, CN 300052 People’s Republic of China; 4 Department of Anaesthesiology, Tianjin First Central Hospital, Fukang Road 24#, Nankai District, Tianjin, CN 300192 People’s Republic of China; 5 Department of Anaesthesiology, Tianjin People’s Hospital Tianjin Union Medical Center, Jieyuan Road 190#, Hongqiao District, Tianjin, CN 300121 People’s Republic of China

**Keywords:** Acute kidney injury, Elderly patients, Hydroxyethyl starch, IL-18, NGAL

## Abstract

**Background:**

Hydroxyethyl starch (HES) is applied to achieve volume expansion during surgery; however, nephrotoxicity may be induced in patients with sepsis. Simultaneously, neutrophil gelatinase-associated lipocalin (NGAL) and IL-18 have been illustrated as pivotal indicators to diagnose the acute kidney injury (AKI) early. This multi-center, randomized, double-blinded, placebo-controlled study aimed to investigate whether 6% HES 130/0.4 administration caused postoperative AKI, which can be revealed by urinary and plasma NGAL and IL-18 estimations in elderly patients with normal renal function undergoing hip arthroplasty under spinal anesthesia.

**Methods:**

120 ASA I–III, patients aged >65 y undergoing hip arthroplasty under spinal anesthesia randomly received 6% HES 130/0.4 or sodium lactate Ringer’s solution 7.5 mL/kg during the first hour of surgery. 118 patients completed the study. Blood pressure, NGAL concentrations, IL18, β_2_ micro-albumin and albumin in urine and creatinine, NGAL and IL-18 in plasma were repeatedly measured before, during, and after surgery.

**Results:**

The groups were balanced in mean arterial pressure, urine and plasma NGAL, plasma IL-18 and creatinine, urine β_2_ microalbumin and albumin (*P* > 0.05). Urine IL-18 was dramatically elevated in both groups after surgery (*P* < 0.05), but did not vary significantly between the groups (*P* > 0.05).

**Conclusion:**

Elderly patients undergoing surgery under spinal anesthesia are a high-risk population in AKI. These patients with normal renal function receiving a spinal anesthesia for a short duration surgery would not develop AKI when 500 mL (small volume) HES is infused.

**Trial registration:**

Identifier: NCT02361736. Registration date was 2 February 2015.

## Background

Hydroxyethyl starch (HES) is infused to sustain circulation in patients undergoing surgery and suffering from trauma and critical disease [1–3]. However, these patients may be subsequently complicated by acute kidney injury (AKI), and HES might effectuate adverse events in renal function. The principal detection of AKI is a rapid increase in plasma creatinine (p-crea) or a sudden drop in urine output [4]. Sex, age, medication, nutrition, and muscle mass exerts a crucial effect on p-crea [5]. Simultaneously, the level of p-crea is upregulated at 24–48 h after renal damage, and hence, the diagnosis of AKI is delayed when p-crea is the sole biomarker for renal injury. Remarkable progress has been made in recent years with respect to achieving an earlier diagnosis of AKI via the evaluation of biomarkers in urine [6]. Neutrophil gelatinase–associated lipocalin (NGAL) is a small protein, filtered via the glomeruli and reabsorbed in the proximal tubules, and thus, the low concentrations can be assessed in the blood and urine [7]. Approximately 6 h after a renal injury, NGAL experiences an abrupt upsurge attributable to an increasing expression and secretion in the epithelial cells of the thick ascending limb of Henle’s loop, the distal tubules, and the collecting ducts [7]. On the other hand, NGAL has been demonstrated and substantiated to predict AKI; malignancies and infections can engender a false increase [8].

Interleukin 18 (IL-18) is an18 kDa cytokine, which is identified as a co-stimulatory factor for the generation of interferon-γ (IFN-γ) in response to toxic shock syndrome. IL-18 shares functional similarities with IL-12, and is synthesized as a 24 kDa precursor molecule without a signal peptide that must be cleaved to produce an active molecule. IL-18 is principally produced from proximal kidney tubules and is a pro-inflammatory factor that can be detected in the urine of earlier AKI animal models. A remarkable upsurge in IL-18 levels in urine during AKI is confirmed in several clinical studies [9–11]. The specificity and susceptibility of IL-18 used in AKI diagnosis is 90%. As a result, IL-18 can be chosen as an effective biomarker to predict AKI.

Intravenous treatment with HES leads to its excretion in urine but is also partially absorbed in the tissues [12, 13]. Several experimental and clinical studies reported that HES molecules were accumulated in the proximal tubule cells with subsequent vacuolization and swelling—a condition known as osmotic nephrosis [14–17]. Recent studies showed that the renal function was impaired when tetrastarch was delivered in patients with sepsis [18, 19]. However, perioperative studies failed to discover the association between AKI occurrence and HES infusion [20–23].

We hypothesized that 6% HES 130/0.4 exerted a nephrotoxic effect in elderly patients, which could be revealed by the assessment and measurements of NGAL and IL-18 levels as a primary outcome [24]. The hemodynamics data, plasma creatinine, urine β_2_ microalbumin, and albumin were the secondary outcomes and also documented. The pharmacokinetic property of Ringer’s lactated solution, different from colloids, cannot contribute to acute kidney injury in clinics [16, 18]; therefore, we selected the infusion of Ringer’s lactate as the control.

## Methods

### Study population

The American Society of Anesthesiologists (ASA) physical status I-III of patients aged >65y scheduled for hip arthroplasty under spinal anesthesia were contacted, informed about the study, and enrolled after written consent was acquired. Exclusion Criteria: any allergy and contraindication to 6% HES 130/0.4; infections and malignancies; sepsis; history of heart failure or NYHA > III; pre-existing renal failure or Cr >108 μmol/L, BUN >8.3 mmol/L; undergoing dialysis treatment; intracranial hemorrhages; long-lasting intake of any non-steroidal anti-inflammatory agent; inability to understand the study information sheet and provide a written consent for participation in the study.

### Intervention

Patients were randomly divided into one of the two groups: (1) patients in group LR received intraoperative placebo lactate Ringer’s at a dose of 7.5 mL/kg; (2) patients in group HES received 6% HES 130/0.4 at a dose of 7.5 mL/kg during the first hour of surgery, followed by administration of 5 mL/kg lactate Ringer’s until the end of the surgery.

### Randomization

The patients were randomly assigned to treatment regimens via a randomization list provided by the Department of Anesthesiology, Tianjin Medical University General Hospital, according to the relevant Standard Operating Procedure (computer-generated random number system). The allocation sequence was concealed until after consent was obtained.

### Blinding

The patients and a treating anesthesiologist involved in the perioperative patient management were incognizant of the group assignment. Primary and secondary outcomes were measured and recorded by another anesthesiologist responsible for data collection, but not involved in the direct treatment of the participating patients and thus, blinded to randomization.

### Procedures and outcomes

All the patients were fasting 12 h before surgery. Upon arrival in the operation theater, the patients were monitored by electrocardiography, pulse oximeter, and noninvasive blood pressure. In addition, a peripheral intravenous line in the left arm and urinary catheter were also attached before anesthesia induction. Subsequent to the collection of the first blood and urine samples, the fluid was infused and mean arterial blood pressure (MAP), heart rate (HR), and arterial saturation were monitored. Then, Cefazolin sodium was injected at prophylactic doses of 2 g in every patient. Spinal anesthesia was administered to the patient in the lateral decubitus position, side of surgery downwards, lumbar puncture preferably midline at L_3_-L_4_ with a pencil point 25-gauge needle. 10 mg Ropivacaine was injected into the subdural space. After 15 min, the patient was maneuvered to a supine. If bradycardia (HR <45 beats/min) and continuous hypotension (MAP <60 mmHg) persisted, additional fluid infusion, atropine (0.5 mg) and phenylephrine (0.1 mg) were also administered. Supplemental oxygen was provided via a nasal cannula. Hemodynamics data was evaluated and recorded at baseline (T_0_), before skin incision (T_1_), at skin incision (T_2_), after skin incision (T_3_), and surgery completion (T_4_). A mean value at every time point was calculated for statistical analysis.

The blood and urine samples were obtained at five time-points: before anesthesia (D_0_), during surgery (D_1_), the first day after surgery (D_2_), the third day after surgery (D_3_), and the fifth day after surgery (D_4_). The postoperative urine and blood samples were collected at 8:00 a.m when patients were fasted, on D_2_, D_3_, and D_4_.

All the urine and blood samples were centrifuged for 10 min at 3500 r/min at 4 °C. Consequently, the samples were maintained at -80 °C until assayed and re-centrifuged before usage in order to minimize any impurities. Every analysis was conducted consecutively by the same laboratory technician to minimize variability in the results.

Creatinine was measured by the sarcosine oxidase method using the commercial kit from Nanjing Jiancheng Bioengineering Institute. The concentration of IL-18 in plasma and urine were assessed by Human IL-18 ELISA Kit (Nanjing Jiancheng Bioengineering Institute). The concentration of NGAL in plasma and urine were measured by Human NGAL ELISA Kit (Nanjing Jiancheng Bioengineering Institute). The urine β_2_ microalbumin and trace albumin were measured by Human β_2_-MG ELISA kit and Human mALB Elisa kit (Nanjing Jiancheng Bioengineering Institute).

The estimated glomerular filtration rate (eGFR) was documented by plasma creatinine through CKD-EPI2009. The 24 h urinary protein quantity was altered by the ratio of urine trace albumin and urine creatinine (ACR). The reference interval of ACR is 0–2.5 for males and 0–3.0 for females.

## Results

**Fig. 1:**
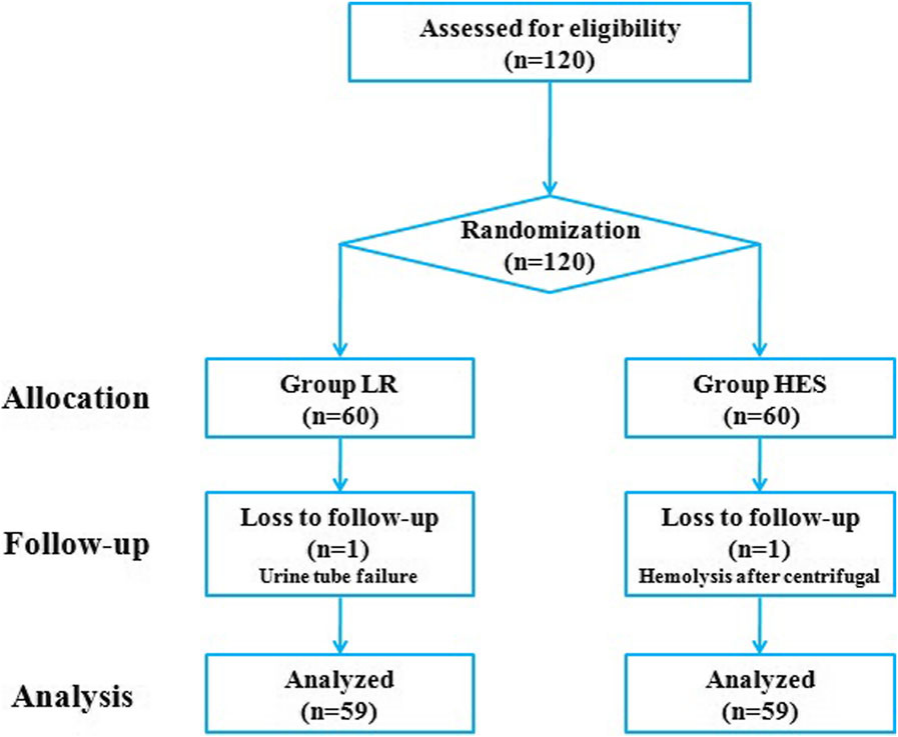
Flow diagram of study

A cohort of 120 patients was included. One patient was excluded before intervention due to urine tube failure. One patient was excluded due to hemolysis after centrifugation. Thus, 59 patients were allocated to the LR group and 59 to the HES group (Fig. 1).

Age, sex, ASA, operation time, and the amount of liquid administered to patients, blood loss, patients needing phenylephrine, phenylephrine dose per patient and length of stay in both the groups were similar (*P* > 0.05, Table 1). The two groups were comparable regarding comorbidities (Table 2). During the span of the entire study, any adverse reactions were not reported.

**Table 1:** Patient characteristics and intraoperative data

Groups	Male/Female	Age (yr)	ASA status (I/II/III)	Operation time (min)	Fluid infusion (ml)	Blood loss (ml)	Patients needing phenylephrine, no.(%)	Phenylephrine dose per patient(mg)	Length of stay (d)
HES	31/28	75.9 ± 7.5	39/15/5	56 ± 4	490 ± 30	100 ± 40	2(3.4)	0.1 ± 0.04	10.5 ± 1.7
LR	29/30	76.4 ± 8.1	37/14/8	58 ± 5	510 ± 20	120 ± 50	5(8.5)	0.15 ± 0.06	11.2 ± 1.1

Values are no. (%) or means(SD)

Patients in group LR received intraoperative placebo Lactate Ringers’ at a dose of 7.5 ml/kg; Patients in group HES received 6% HES 130/0.4 at a dose of 7.5 ml/kg in the first hour of surgery, and then, Lactate Ringers’ was administrated 5 ml/kg until the end of the surgery. All data were presented as mean ± SD or number of patients. *ASA* American Society of Anaesthesiologists, *HES* 6% hydroxyethyl starch 130/0.4, *LR* Lactate Ringers

**Table 2:** Patients’ comorbidities

Comorbidities	HES(*n* = 59)	LR(*n* = 59)
Hypertension,no.(%)	49(83.0)	51(86.4)
Diabetes mellitus(typeII),no.(%)	12(20.3)	10(16.9)
Stroke,no.(%)	1(1.7)	2(3.4)
COPD,no.(%)	1(1.7)	3(5.1)
CABG,no.(%)	2(3.4)	2(3.4)

*COPD* chronic obstructive pulmonary disease, *CABG* coronary artery bypass graft, *HES* 6% hydroxyethyl starch 130/0.4, *LR* Lactate Ringers’

Values are no. (%)

A significant difference of MAP and HR between T_0_ and T_2-4_ was not observed in either of the groups (*P* > 0.05, Table 3). When compared with D_0_, the concentration of IL-18 in urine was dramatically enhanced in both the groups on D_2_, D_3_, and D_4_ (*P* < 0.05, Table 4); however, no significant difference was seen between the two groups. Similarly, both groups showed a balanced plasma IL-18 at every time-point (*P* > 0.05, Table 4).

**Table 3:** Intraoperative mean arterial blood pressure and heart rate

Groups	MAP(mmHg)					HR(beats/min)				
	T_0_	T_1_	T_2_	T_3_	T_4_	T_0_	T_1_	T_2_	T_3_	T_4_
HES	112 ± 7	105 ± 6	99 ± 8	100 ± 6	101 ± 7	65 ± 3	68 ± 5	66 ± 4	67 ± 5	63 ± 4
LR	109 ± 5	106 ± 8	98 ± 6	96 ± 7	99 ± 6	70 ± 2	69 ± 3	71 ± 5	66 ± 6	65 ± 3

Patients in group LR received intraoperative placebo Lactate Ringers’ at a dose of 7.5 ml/kg; Patients in group HES received 6% HES 130/0.4 at a dose of 7.5 ml/kg in the first hour of surgery, and then, Lactate Ringers’ was administrated 5 ml/kg until the end of the surgery. Haemodynamics data was evaluated and recorded at baseline (T_0_), before skin incision (T_1_), skin incision (T_2_), after skin incision (T_3_), surgery completed (T_4_). All data were presented as mean ± SD. *HES* 6% hydroxyethyl starch 130/0.4, *LR* Lactate Ringers’, *MAP* Mean arterial pressure

**Table 4:** Renal function

Groups	D_0_	D_1_	D_2_	D_3_	D_4_
p-NGAL (ng/ml)					
HES	4.0 ± 0.3	4.2 ± 0.5	4.3 ± 0.5	4.2 ± 0.5	4.4 ± 0.6
LR	4.1 ± 0.8	4.0 ± 0.7	4.4 ± 0.9	4.1 ± 0.7	4.3 ± 0.8
u-NGAL (ng/ml)					
HES	4.9 ± 0.8	5.1 ± 0.3	4.9 ± 0.4	5.2 ± 0.5	5.1 ± 0.4
LR	5.6 ± 0.7	5.2 ± 0.4	4.9 ± 0.3	5.0 ± 0.3	5.1 ± 0.3
p-IL18 (μg/l)					
HES	79.3 ± 9.8	81.0 ± 10.4	84.2 ± 13.8	83.4 ± 10.2	85.1 ± 11.3
LR	81.0 ± 14.5	81.4 ± 9.0	83.8 ± 12.5	86.5 ± 14.1	81.5 ± 6.1
u-IL-18 (μg/l)					
HES	82.7 ± 8.5	89.5 ± 5.8	109.6 ± 9.2^*^	140.5 ± 10.9^*^	159.7 ± 16.9^*^
LR	86.6 ± 8.5	83.2 ± 4.8	118.5 ± 11.4^*^	142.6 ± 18.1^*^	162.0 ± 18.5^*^
eGFR (ml/min/1.73 m^2^)					
HES	63.9 ± 14.3	63.5 ± 13.5	64 ± 14.7	72.8 ± 10.4	68.6 ± 12.4
LR	61.9 ± 8.3	63.5 ± 9.6	63 ± 6.2	64 ± 9.4	66.4 ± 9.2
ACR					
HES	1.96 ± 0.21	1.98 ± 0.19	1.90 ± 0.09	1.99 ± 0.13	1.95 ± 0.20
LR	1.89 ± 0.14	1.84 ± 0.20	1.90 ± 0.19	1.94 ± 0.15	1.92 ± 0.12
u-β2 micro-albumin (mg/l)					
HES	96.4 ± 11.2	97.1 ± 12.2	101.7 ± 9.4	99.4 ± 15.7	103.0 ± 13.8
LR	93.2 ± 8.9	90.6 ± 8.2	99.8 ± 14.2	104.7 ± 15.0	93.2 ± 12.3
p-Creatinine (umol/l)					
HES	92.5 ± 9.3	94.6 ± 7.9	93.1 ± 9.1	81.2 ± 8.6	85.9 ± 7.7
LR	85.7 ± 5.9	86 ± 9.1	85.3 ± 6.3	85.1 ± 7.9	82.6 ± 9.2
u-Albumin (g/l)					
HES	87.4 ± 4.1	90.2 ± 4.3	91.1 ± 5.1	96.1 ± 6.3	97.5 ± 7.0
LR	86.6 ± 5.7	87.1 ± 2.8	92.3 ± 3.8	97 ± 4.7	98.1 ± 6.4

Patients in group LR received intraoperative placebo Lactate Ringers’ at a dose of 7.5 ml/kg; Patients in group HES received 6% HES 130/0.4 at a dose of 7.5 ml/kg in the first hour of surgery, and then, Lactate Ringers’ was administrated 5 ml/kg until the end of the surgery. Blood and urine sample were got at five time points: before anaesthesia (D_0_), during surgery (D_1_), the first day after surgery (D_2_), the third day after surgery (D_3_), the fifth day after surgery (D_4_). ACR, eGFR, urine (u) and plasma (p) NGAL, plasma creatinine, urine β_2_ micro-albumin and albumin were assessed. Values are presented as mean ± SD, *P < 0.05 vs. D_0_

*HES* 6% hydroxyethyl starch 130/0.4, *LR* Lactate Ringers’, *u*-*NGAL* urine Neutrophil gelatinase–associated lipocalin, *p*-*NGAL* plasma Neutrophil gelatinase–associated lipocalin, *eGFR* estimated glomerular filtration rate, *ACR* ratio of urine trace albumin and urine creatinine

A remarkable alteration in the levels of eGFR, ACR, urine and plasma NGAL, plasma creatinine, urine β_2_ microalbumin, and trace albumin was not noted in both groups (*P* > 0.05, Table 4).

## Discussion

In the current study, we investigated the influence of intraoperative administration of lactate Ringer’s and 6% HES 130/0.4 on elderly patients with normal renal function undergoing hip arthroplasty under spinal anesthesia. The objective of our study was to illustrate whether 6% HES 130/0.4 infusion played a nephrotoxic role that can be detected via blood pressure and concentrations of NGAL, IL18, β_2_ microalbumin and albumin in urine and creatinine, as well as, NGAL and IL-18 in plasma. The elderly patients with normal renal function who underwent hip arthroplasty with spinal anesthesia demonstrated that intraoperative HES (500 mL) infusion did not cause postoperative AKI.

A recent study suggested that utilizing HES may elicit AKI in patients with sepsis. The current study selected elderly patients with normal renal function to accept spinal anesthesia and excluded the patients with infection, malignant tumors, and other factors that may affect the level of NGAL and IL-18 in plasma and urine. We discovered that urine IL-18 was increased significantly and similarly in both groups post-surgery, indicating that elderly patients are a high-risk population for the development of AKI. Furthermore, HES is not implicated in this process. The phenomenon in elderly patients of not developing AKI from the infusion of 6% HES 130/0.4 in our study might be attributed to the short duration of surgery and the small volume of HES infusion. A previous study suggested that AKI occurred after 36 h when septic patients received more than 2000 mL HES [19]. The most dramatical difference between the recent multi-center studies (CRYSTMAS, 6S, and CHEST) and the current investigation is the patients’ characteristics. The CHEST study by Myburgh et al. included patients with signs of hypovolemia, the CRYSTMAS study by Guidet et al. and the 6S trial by Perner et al. included patients who suffered from clinically defined severe sepsis. Furthermore, the vast majority of the patients in these three studies were medical patients [18, 19, 25]. Contrastingly, the present study exclusively investigated elderly patients with normal kidney function. Thus, we considered that 500 mL HES would not contribute to AKI in elderly patients who required undergoing hip arthroplasty with spinal anesthesia.

IL-18 has been manifested as an indicator for the early prediction of AKI. Urinary IL-18 was considerably elevated for the diagnosis of AKI with sensitivity and specificity of 90%. To our knowledge, this is the first study to discover that urine IL-18 levels were significantly increased in elderly patients undergoing hip arthroplasty under the influence of spinal anesthesia. On the other hand, other indicators such as eGFR, ACR, plasma creatinine, blood and urine NGAL, and urine β_2_ microglobulin levels did not show a remarkable alteration. According to the KDIGO AKI classification (the KDIGO 1 stage is a minimum increase in plasma creatinine concentration >50% or an increase in 27 μmol/L of creatinine within 48 h), all the patients in this study could not be diagnosed as AKI. However, we are convinced that the postoperative increase of urine IL-18 levels might be associated with mild renal tubular damage. Importantly, a significant difference in the urine level of IL-18 between the two groups was not observed, indicating that HES exposure is not the key reason of AKI in elderly patients.

Clinical studies have reported that NGAL production is a result of a marked immune response in the event of AKI. Simultaneously, NGAL is an independent predictor of AKI after sepsis, ischemia, or renal toxicity. Additionally, IL-18 has been identified as specific in ischemic AKI. We also found that IL-18 but not NGAL and other indicators experienced a rising trend, suggesting that the postoperative kidney damage may be due to the ischemic effect of spinal anesthesia. Thus, according to results, perioperative elderly patients are at high risk of ischemic AKI, and risk of ischemia should be avoided in such patients.

Although IL-18 was profoundly increased in both groups, it was not a sharp rise, suggesting that all the patients in the current study developed a mild renal inflammatory response potentially arising as a consequence of the surgery or anesthesia. This mild inflammatory response raised the level of IL-18 in urine, thereby increasing the load of glomerular filtration rate. Only when the concentration of plasma IL-18 attains a specific threshold, AKI would be generated [26]. This may also explain why the patients in our study did not develop AKI, and the other indicators were not significantly increased, including NGAL.

One possible limitation of the present study is that only the end of the operation and 1, 3, and 5 days after operation were selected as the assessment time-points according to the time of AKI diagnosis. A further follow-up study on the minimal renal injury was lacking, and thus, we could not determine when the concentration of urine IL-18 decreased to baseline levels [24]. In the future, additional clinical studies with a larger volume of HES and more time-points will be essential to substantiate the conclusion of the present study.

## Conclusion

In summary, the present data asserts that elderly patients with hip arthroplasty under spinal anesthesia are a high-risk population for AKI, and the risk of ischemia should be avoided. Furthermore, elderly patients with normal renal function receiving a spinal anesthesia for a short duration surgery would not develop AKI when a small amount of HES (500 mL) was infused. Yet, caution must be exercised in translating these results to an average clinical situation.

## Abbreviations

ACR: Urine trace albumin/urine creatinine ratio
AKI: Acute kidney injury
eGFR: Estimated glomerular filtration rate
HES: Hydroxyethyl starch 130/0.4
LR: Lactate Ringer’s
NGAL: Neutrophil gelatinase-associated lipocalin
